# *N*-(2,3-Dichloro­phen­yl)-2-nitro­benzene­sulfonamide

**DOI:** 10.1107/S1600536812048076

**Published:** 2012-12-12

**Authors:** U. Chaithanya, Sabine Foro, B. Thimme Gowda

**Affiliations:** a Department of Chemistry, Mangalore University, Mangalagangotri 574 199, Mangalore, India; b Institute of Materials Science, Darmstadt University of Technology, Petersenstrasse 23, D-64287 Darmstadt, Germany

## Abstract

In the title compound, C_12_H_8_Cl_2_N_2_O_4_S, the N—C bond in the C—SO_2_—NH—C segment has *gauche* torsions with respect to the S=O bonds. Further, the N—H bond is *syn* to the *ortho*-nitro group in the sulfonyl benzene ring and also *syn* to both the *ortho*- and *meta*-Cl atoms in the aniline ring. The mol­ecule is twisted at the S—N bond with a torsion angle of 61.15 (18)°. The dihedral angle between the planes of the benzene rings is 68.00 (6)°. The amide H atom exhibits an intra­molecular bifurcated N—H⋯(O,O) hydrogen bond. In the crystal, pairs of N—H⋯O(S) hydrogen bonds link the mol­ecules into inversion dimers with *R*_2_^2^(8) motifs.

## Related literature

For our studies on the effects of substituents on the structures and other aspects of *N*-(ar­yl)-amides, see: Gowda & Weiss (1994 ▶), of *N*-aryl­sulfonamides, see: Chaithanya *et al.* (2012 ▶); Gowda *et al.* (2002 ▶) and of *N*-chloroaryl-sulfonamides, see: Gowda & Shetty (2004 ▶); Shetty & Gowda (2004 ▶).
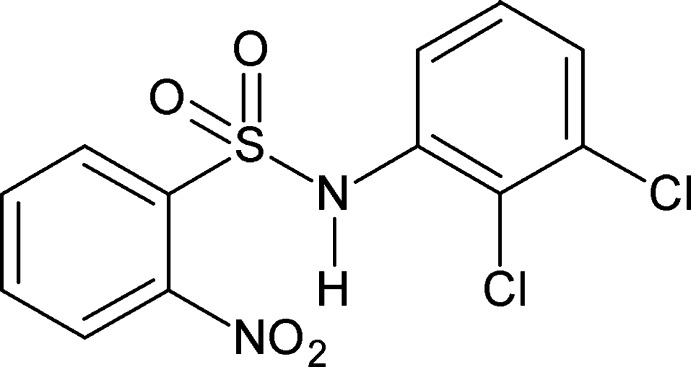


## Experimental

### 

#### Crystal data

C_12_H_8_Cl_2_N_2_O_4_S
*M* *_r_* = 347.16
Monoclinic, 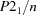

*a* = 8.2197 (5) Å
*b* = 15.863 (1) Å
*c* = 11.0108 (6) Å
β = 93.450 (6)°
*V* = 1433.09 (15) Å^3^
*Z* = 4
Mo *K*α radiation
μ = 0.61 mm^−1^
*T* = 293 K
0.44 × 0.36 × 0.28 mm

#### Data collection

Oxford Diffraction Xcalibur diffractometer with a Sapphire CCD detector
Absorption correction: multi-scan (*CrysAlis RED*; Oxford Diffraction, 2009 ▶) *T*_min_ = 0.774, *T*_max_ = 0.847
5760 measured reflections
2923 independent reflections
2304 reflections with *I* > 2σ(*I*)
*R*_int_ = 0.013

#### Refinement

*R*[*F* ^2^ > 2σ(*F* ^2^)] = 0.037
*wR*(*F* ^2^) = 0.092
*S* = 1.01
2923 reflections
193 parameters
2 restraints
H atoms treated by a mixture of independent and constrained refinement
Δρ_max_ = 0.35 e Å^−3^
Δρ_min_ = −0.34 e Å^−3^

### 

Data collection: *CrysAlis CCD* (Oxford Diffraction, 2009 ▶); cell refinement: *CrysAlis CCD*; data reduction: *CrysAlis RED* (Oxford Diffraction, 2009 ▶); program(s) used to solve structure: *SHELXS97* (Sheldrick, 2008 ▶); program(s) used to refine structure: *SHELXL97* (Sheldrick, 2008 ▶); molecular graphics: *PLATON* (Spek, 2009 ▶); software used to prepare material for publication: *SHELXL97*.

## Supplementary Material

Crystal structure: contains datablock(s) I, global. DOI: 10.1107/S1600536812048076/rz5027sup1.cif

Structure factors: contains datablock(s) I. DOI: 10.1107/S1600536812048076/rz5027Isup2.hkl

Supplementary material file. DOI: 10.1107/S1600536812048076/rz5027Isup3.cml

Additional supplementary materials:  crystallographic information; 3D view; checkCIF report

## Figures and Tables

**Table 1:** Hydrogen-bond geometry (Å, °)

*D*—H⋯*A*	*D*—H	H⋯*A*	*D*⋯*A*	*D*—H⋯*A*
N1—H1*N*⋯O2^i^	0.83 (2)	2.48 (2)	3.136 (2)	137 (2)
N1—H1*N*⋯O3	0.83 (2)	2.51 (2)	3.065 (3)	125 (2)

Symmetry code: (i) 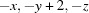.

**Fig. 1.**
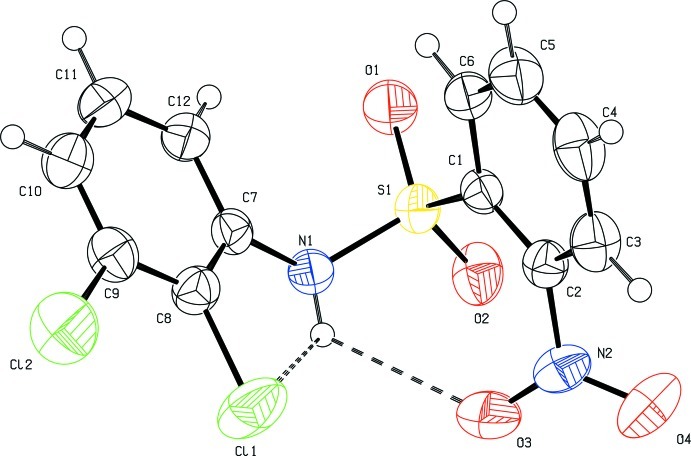
The molecular structure of the title compound, showing the atom labelling scheme and with displacement ellipsoids drawn at the 50% probability level.

**Fig. 2.**
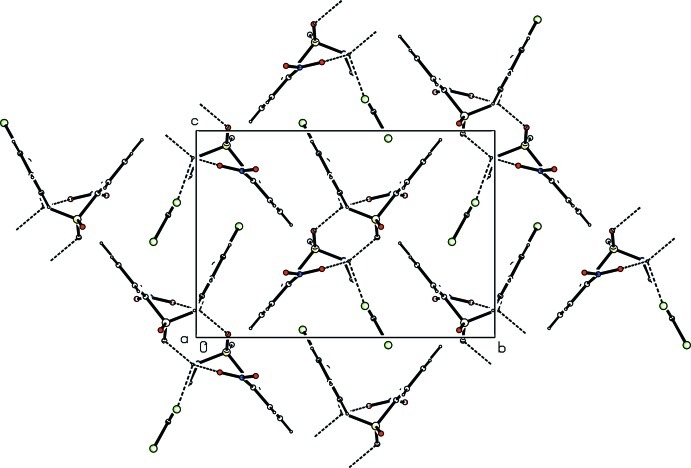
Molecular packing of the title compound viewed down the a axis with hydrogen bonding shown as dashed lines.

## supplementary crystallographic information

### Comment

As a part of studying the effect of substituents on the structures and other aspects of *N*-(aryl)-amides (Gowda *et al.*, 1994); *N*-arylsulfonamides (Chaithanya *et al.*, 2012; Gowda *et al.*, 2002) and *N*-chloroarylsulfonamides(Gowda & Shetty, 2004; Shetty & Gowda, 2004), in the present work, the crystal structure of *N*-(2,3-dichlorophenyl)-2-nitrobenzenesulfonamide (I) has been determined (Fig. 1). The conformation of the N—H bond is *syn* to the *ortho*-nitro group in the sulfonyl benzene ring and also *syn* to both the *ortho*- and *meta*-Cl atoms in the anilino ring, compared to the *syn* conformation between the N—H bond and the *ortho*-nitro group in the sulfonyl benzene ring and *anti* conformation between the N—H bond and the *ortho*- and *meta*-methyl groups in the anilino ring observed in *N*-(2,3-dimethylphenyl)-2-nitrobenzenesulfonamide (II) (Chaithanya *et al.*, 2012).

The molecule in (I) is twisted at the S—N bond with the torsion angle of 61.15 (18)°, compared to the values of -60.37 (30) and 58.81 (34)° in the two independent molecules of (II).

The dihedral angle between the sulfonyl and the anilino ring is 68.00 (6)°, compared to the values of 53.67 (8) and 56.99 (9)° in the two molecules of (II).

The amide H-atom showed bifurcated intramolecular H-bonding with the O-atom of the *ortho*-nitro group in the sulfonyl benzene ring, generating S(7) motifs and the intermolecular H-bonding with the sulfonyl oxygen atom of the other molecule, generating inversion dimers (Table 1, Fig. 2.)

### Experimental

The title compound was prepared by treating 2-nitrobenzenesulfonylchloride with 2,3-dichloroaniline in the stoichiometric ratio and boiling the reaction mixture for 15 minutes. The reaction mixture was then cooled to room temperature and added to ice cold water (100 ml). The resultant solid *N*-(2,3-dichlorophenyl)-2-nitrobenzenesulfon- amide was filtered under suction and washed thoroughly with cold water and dilute HCl to remove the excess sulfonylchloride and aniline, respectively. It was then recrystallized to constant melting point from dilute ethanol. The purity of the compound was checked and characterized by its infrared spectra.

Prism like colourless single crystals of the title compound used in X-ray diffraction studies were grown in ethanolic solution by slow evaporation of the solvent at room temperature.

### Refinement

H atoms bonded to C were positioned with idealized geometry using a riding model with the aromatic C—H = 0.93 Å. The amino H atom was freely refined with the N—H distance restrained to 0.86 (2) Å. All H atoms were refined with isotropic displacement parameters set at 1.2 *U*_eq_ of the parent atom.

### Crystal data

**Table:**

C_12_H_8_Cl_2_N_2_O_4_S	*F*(000) = 704
*M**_r_* = 347.16	*D*_x_ = 1.609 Mg m^−^^3^
Monoclinic, *P*2_1_/*n*	Mo *K*α radiation, λ = 0.71073 Å
Hall symbol: -P 2yn	Cell parameters from 2186 reflections
*a* = 8.2197 (5) Å	θ = 2.6–27.7°
*b* = 15.863 (1) Å	µ = 0.61 mm^−^^1^
*c* = 11.0108 (6) Å	*T* = 293 K
β = 93.450 (6)°	Prism, colourless
*V* = 1433.09 (15) Å^3^	0.44 × 0.36 × 0.28 mm
*Z* = 4	

### Data collection

**Table:**

Oxford Diffraction Xcalibur diffractometer with a Sapphire CCD detector	2923 independent reflections
Radiation source: fine-focus sealed tube	2304 reflections with *I* > 2σ(*I*)
Graphite monochromator	*R*_int_ = 0.013
Rotation method data acquisition using ω scans	θ_max_ = 26.4°, θ_min_ = 2.6°
Absorption correction: multi-scan (*CrysAlis RED*; Oxford Diffraction, 2009)	*h* = −4→10
*T*_min_ = 0.774, *T*_max_ = 0.847	*k* = −19→19
5760 measured reflections	*l* = −13→13

### Refinement

**Table:**

Refinement on *F*^2^	Primary atom site location: structure-invariant direct methods
Least-squares matrix: full	Secondary atom site location: difference Fourier map
*R*[*F*^2^ > 2σ(*F*^2^)] = 0.037	Hydrogen site location: inferred from neighbouring sites
*wR*(*F*^2^) = 0.092	H atoms treated by a mixture of independent and constrained refinement
*S* = 1.01	*w* = 1/[σ^2^(*F*_o_^2^) + (0.0395*P*)^2^ + 0.7218*P*] where *P* = (*F*_o_^2^ + 2*F*_c_^2^)/3
2923 reflections	(Δ/σ)_max_ = 0.002
193 parameters	Δρ_max_ = 0.35 e Å^−^^3^
2 restraints	Δρ_min_ = −0.34 e Å^−^^3^

### Special details

**Table:**

Experimental. Absorption correction: CrysAlis RED (Oxford Diffraction, 2009) Empirical absorption correction using spherical harmonics, implemented in SCALE3 ABSPACK scaling algorithm.
Geometry. All e.s.d.'s (except the e.s.d. in the dihedral angle between two l.s. planes) are estimated using the full covariance matrix. The cell e.s.d.'s are taken into account individually in the estimation of e.s.d.'s in distances, angles and torsion angles; correlations between e.s.d.'s in cell parameters are only used when they are defined by crystal symmetry. An approximate (isotropic) treatment of cell e.s.d.'s is used for estimating e.s.d.'s involving l.s. planes.
Refinement. Refinement of *F*^2^ against ALL reflections. The weighted *R*-factor *wR* and goodness of fit *S* are based on *F*^2^, conventional *R*-factors *R* are based on *F*, with *F* set to zero for negative *F*^2^. The threshold expression of *F*^2^ > σ(*F*^2^) is used only for calculating *R*-factors(gt) *etc*. and is not relevant to the choice of reflections for refinement. *R*-factors based on *F*^2^ are statistically about twice as large as those based on *F*, and *R*- factors based on ALL data will be even larger.

### Fractional atomic coordinates and isotropic or equivalent isotropic displacement parameters (Å^2^)

**Table:**

	*x*	*y*	*z*	*U*_iso_*/*U*_eq_	
C1	0.1808 (2)	0.83051 (12)	0.19670 (17)	0.0339 (4)	
C2	0.0312 (2)	0.80943 (13)	0.24194 (17)	0.0376 (4)	
C3	0.0199 (3)	0.75308 (15)	0.3365 (2)	0.0494 (6)	
H3	−0.0814	0.7382	0.3631	0.059*	
C4	0.1601 (3)	0.71913 (15)	0.3910 (2)	0.0572 (6)	
H4	0.1538	0.6820	0.4560	0.069*	
C5	0.3075 (3)	0.73969 (15)	0.3501 (2)	0.0576 (6)	
H5	0.4017	0.7171	0.3883	0.069*	
C6	0.3199 (3)	0.79411 (14)	0.2517 (2)	0.0455 (5)	
H6	0.4215	0.8060	0.2229	0.055*	
C7	0.3264 (2)	1.02271 (12)	0.21739 (18)	0.0352 (4)	
C8	0.2745 (3)	1.05973 (14)	0.32373 (19)	0.0410 (5)	
C9	0.3880 (3)	1.09390 (15)	0.40794 (19)	0.0474 (5)	
C10	0.5515 (3)	1.08976 (16)	0.3900 (2)	0.0573 (6)	
H10	0.6272	1.1122	0.4473	0.069*	
C11	0.6023 (3)	1.05199 (16)	0.2863 (2)	0.0593 (7)	
H11	0.7131	1.0487	0.2742	0.071*	
C12	0.4916 (3)	1.01906 (14)	0.2003 (2)	0.0459 (5)	
H12	0.5280	0.9942	0.1304	0.055*	
N1	0.2103 (2)	0.99427 (11)	0.12555 (16)	0.0385 (4)	
H1N	0.116 (2)	1.0115 (14)	0.131 (2)	0.046*	
N2	−0.1233 (2)	0.84663 (14)	0.19410 (17)	0.0477 (5)	
O1	0.37021 (19)	0.88086 (10)	0.03520 (14)	0.0518 (4)	
O2	0.0757 (2)	0.89329 (10)	−0.01537 (13)	0.0526 (4)	
O3	−0.1264 (2)	0.92105 (13)	0.16889 (19)	0.0696 (5)	
O4	−0.2424 (2)	0.80065 (14)	0.18634 (18)	0.0736 (6)	
Cl1	0.06982 (8)	1.06521 (6)	0.34758 (7)	0.0781 (3)	
Cl2	0.32597 (10)	1.14330 (5)	0.53734 (6)	0.0784 (3)	
S1	0.21072 (6)	0.89869 (3)	0.07156 (4)	0.03673 (14)	

### Atomic displacement parameters (Å^2^)

**Table:**

	*U*^11^	*U*^22^	*U*^33^	*U*^12^	*U*^13^	*U*^23^
C1	0.0374 (10)	0.0298 (10)	0.0343 (9)	0.0005 (8)	−0.0010 (8)	−0.0022 (8)
C2	0.0400 (11)	0.0381 (11)	0.0346 (10)	−0.0017 (9)	0.0028 (9)	−0.0032 (8)
C3	0.0596 (14)	0.0482 (13)	0.0414 (12)	−0.0091 (11)	0.0102 (11)	0.0017 (10)
C4	0.0835 (15)	0.0432 (13)	0.0435 (12)	−0.0079 (13)	−0.0078 (12)	0.0094 (10)
C5	0.0628 (13)	0.0449 (14)	0.0621 (15)	0.0044 (11)	−0.0222 (11)	0.0079 (11)
C6	0.0398 (11)	0.0391 (12)	0.0563 (13)	0.0009 (9)	−0.0080 (10)	0.0007 (10)
C7	0.0371 (10)	0.0286 (10)	0.0397 (10)	−0.0019 (8)	0.0003 (8)	0.0034 (8)
C8	0.0383 (11)	0.0430 (12)	0.0421 (11)	−0.0025 (9)	0.0058 (9)	0.0006 (9)
C9	0.0555 (14)	0.0459 (13)	0.0405 (11)	−0.0005 (11)	0.0010 (10)	−0.0066 (10)
C10	0.0496 (14)	0.0566 (16)	0.0637 (15)	−0.0016 (12)	−0.0136 (12)	−0.0145 (12)
C11	0.0360 (12)	0.0609 (16)	0.0803 (18)	−0.0010 (11)	−0.0013 (12)	−0.0143 (14)
C12	0.0404 (11)	0.0445 (13)	0.0531 (13)	−0.0006 (10)	0.0059 (10)	−0.0082 (10)
N1	0.0361 (9)	0.0356 (10)	0.0431 (9)	0.0017 (8)	−0.0044 (8)	0.0006 (8)
N2	0.0357 (10)	0.0632 (14)	0.0450 (10)	0.0018 (9)	0.0068 (8)	−0.0006 (9)
O1	0.0524 (9)	0.0535 (10)	0.0518 (9)	−0.0029 (8)	0.0213 (8)	−0.0098 (7)
O2	0.0637 (10)	0.0515 (10)	0.0406 (8)	−0.0080 (8)	−0.0149 (7)	0.0003 (7)
O3	0.0492 (10)	0.0597 (12)	0.0991 (14)	0.0157 (9)	−0.0011 (10)	0.0076 (11)
O4	0.0416 (9)	0.0996 (16)	0.0790 (13)	−0.0178 (10)	−0.0019 (9)	0.0072 (11)
Cl1	0.0441 (4)	0.1163 (7)	0.0756 (5)	−0.0079 (4)	0.0186 (3)	−0.0316 (4)
Cl2	0.0881 (5)	0.0950 (6)	0.0527 (4)	−0.0046 (4)	0.0083 (4)	−0.0295 (4)
S1	0.0411 (3)	0.0379 (3)	0.0312 (2)	−0.0025 (2)	0.0018 (2)	−0.0019 (2)

### Geometric parameters (Å, º)

**Table:**

C1—C6	1.387 (3)	C8—C9	1.386 (3)
C1—C2	1.395 (3)	C8—Cl1	1.721 (2)
C1—S1	1.780 (2)	C9—C10	1.371 (3)
C2—C3	1.379 (3)	C9—Cl2	1.730 (2)
C2—N2	1.469 (3)	C10—C11	1.376 (4)
C3—C4	1.376 (3)	C10—H10	0.9300
C3—H3	0.9300	C11—C12	1.377 (3)
C4—C5	1.358 (4)	C11—H11	0.9300
C4—H4	0.9300	C12—H12	0.9300
C5—C6	1.394 (3)	N1—S1	1.6287 (18)
C5—H5	0.9300	N1—H1N	0.827 (16)
C6—H6	0.9300	N2—O3	1.213 (3)
C7—C12	1.384 (3)	N2—O4	1.220 (2)
C7—C8	1.399 (3)	O1—S1	1.4221 (16)
C7—N1	1.422 (3)	O2—S1	1.4236 (16)
			
C6—C1—C2	117.74 (19)	C10—C9—C8	120.7 (2)
C6—C1—S1	116.24 (16)	C10—C9—Cl2	118.70 (18)
C2—C1—S1	126.01 (15)	C8—C9—Cl2	120.55 (18)
C3—C2—C1	121.7 (2)	C9—C10—C11	119.3 (2)
C3—C2—N2	115.78 (19)	C9—C10—H10	120.4
C1—C2—N2	122.49 (18)	C11—C10—H10	120.4
C4—C3—C2	119.4 (2)	C12—C11—C10	121.0 (2)
C4—C3—H3	120.3	C12—C11—H11	119.5
C2—C3—H3	120.3	C10—C11—H11	119.5
C5—C4—C3	120.1 (2)	C11—C12—C7	120.3 (2)
C5—C4—H4	119.9	C11—C12—H12	119.9
C3—C4—H4	119.9	C7—C12—H12	119.9
C4—C5—C6	121.0 (2)	C7—N1—S1	122.60 (14)
C4—C5—H5	119.5	C7—N1—H1N	115.9 (16)
C6—C5—H5	119.5	S1—N1—H1N	110.9 (17)
C1—C6—C5	120.0 (2)	O3—N2—O4	124.1 (2)
C1—C6—H6	120.0	O3—N2—C2	118.64 (19)
C5—C6—H6	120.0	O4—N2—C2	117.2 (2)
C12—C7—C8	118.85 (19)	O1—S1—O2	119.54 (10)
C12—C7—N1	120.83 (19)	O1—S1—N1	108.08 (10)
C8—C7—N1	120.21 (18)	O2—S1—N1	106.41 (9)
C9—C8—C7	119.83 (19)	O1—S1—C1	105.65 (10)
C9—C8—Cl1	120.22 (17)	O2—S1—C1	110.21 (9)
C7—C8—Cl1	119.92 (16)	N1—S1—C1	106.24 (9)
			
C6—C1—C2—C3	−1.3 (3)	Cl2—C9—C10—C11	−178.8 (2)
S1—C1—C2—C3	177.24 (17)	C9—C10—C11—C12	0.5 (4)
C6—C1—C2—N2	177.51 (19)	C10—C11—C12—C7	−0.6 (4)
S1—C1—C2—N2	−4.0 (3)	C8—C7—C12—C11	−0.5 (3)
C1—C2—C3—C4	2.6 (3)	N1—C7—C12—C11	175.6 (2)
N2—C2—C3—C4	−176.3 (2)	C12—C7—N1—S1	57.5 (3)
C2—C3—C4—C5	−1.4 (4)	C8—C7—N1—S1	−126.45 (18)
C3—C4—C5—C6	−1.0 (4)	C3—C2—N2—O3	138.7 (2)
C2—C1—C6—C5	−1.1 (3)	C1—C2—N2—O3	−40.2 (3)
S1—C1—C6—C5	−179.79 (18)	C3—C2—N2—O4	−38.8 (3)
C4—C5—C6—C1	2.3 (4)	C1—C2—N2—O4	142.3 (2)
C12—C7—C8—C9	1.7 (3)	C7—N1—S1—O1	−51.85 (19)
N1—C7—C8—C9	−174.38 (19)	C7—N1—S1—O2	178.59 (16)
C12—C7—C8—Cl1	179.99 (17)	C7—N1—S1—C1	61.15 (18)
N1—C7—C8—Cl1	3.9 (3)	C6—C1—S1—O1	16.35 (18)
C7—C8—C9—C10	−1.9 (3)	C2—C1—S1—O1	−162.21 (17)
Cl1—C8—C9—C10	179.9 (2)	C6—C1—S1—O2	146.80 (16)
C7—C8—C9—Cl2	177.68 (17)	C2—C1—S1—O2	−31.8 (2)
Cl1—C8—C9—Cl2	−0.6 (3)	C6—C1—S1—N1	−98.31 (17)
C8—C9—C10—C11	0.7 (4)	C2—C1—S1—N1	83.13 (19)

### Hydrogen-bond geometry (Å, º)

**Table:**

*D*—H···*A*	*D*—H	H···*A*	*D*···*A*	*D*—H···*A*
N1—H1*N*···O2^i^	0.83 (2)	2.48 (2)	3.136 (2)	137 (2)
N1—H1*N*···O3	0.83 (2)	2.51 (2)	3.065 (3)	125 (2)
N1—H1*N*···Cl1	0.83 (2)	2.58 (2)	2.9858 (19)	111 (2)

Symmetry code: (i) −*x*, −*y*+2, −*z*.
